# Evaluation of the FACSPresto, a New Point of Care Device for the Enumeration of CD4% and Absolute CD4+ T Cell Counts in HIV Infection

**DOI:** 10.1371/journal.pone.0157546

**Published:** 2016-07-07

**Authors:** Azure Tariro Makadzange, Carola Bogezi, Kathryn Boyd, Anesu Gumbo, Dorinda Mukura, Allen Matubu, Chiratidzo Ellen Ndhlovu

**Affiliations:** 1 Ragon Institute of MGH, MIT and Harvard, Cambridge, Massachusetts, United States of America; 2 Department of Medicine, University of Zimbabwe College of Health Sciences, Harare, Zimbabwe; 3 Department of Obstetrics and Gynecology, UZ-UCSF Collaborative Project, University of Zimbabwe College of Health Sciences, Harare, Zimbabwe; China Medical University, TAIWAN

## Abstract

**Introduction:**

Enumeration of CD4+ T lymphocytes is important for pre-ART disease staging and screening for opportunistic infections, however access to CD4 testing in resource limited settings is poor. Point of care (POC) technologies can facilitate improved access to CD4 testing. We evaluated the analytical performance of a novel POC device the FACSPresto compared to the FACSCalibur as a reference standard and to the PIMA, a POC device in widespread use in sub-Saharan Africa.

**Method:**

Specimens were obtained from 253 HIV infected adults. Venous blood samples were analyzed on the FACSPresto and the FACSCalibur, in a subset of 41 samples additional analysis was done on the PIMA.

**Results:**

The absolute CD4 count results obtained on the FACSPresto were comparable to those on the FACSCalibur with low absolute (9.5cells/μl) and relative bias (3.2%). Bias in CD4% values was also low (1.06%) with a relative bias of 4.9%. The sensitivity was lower at a CD4 count threshold of ≤350cells/μl compared with ≤500cells/μl (84.9% vs. 92.8%) resulting in a high upward misclassification rate at low CD4 counts. Specificity at thresholds of ≤350cells/μl and ≤500cells/μl were 96.6% and 96.8% respectively. The PIMA had a high absolute (-68.6cells/μl) and relative bias (-10.5%) when compared with the FACSCalibur. At thresholds of ≤350cells/μl and ≤500cells/μl the sensitivity was 100% and 95.5% respectively; specificity was 85.7% and 84.2% respectively. The coefficients of repeatability were 4.13%, 5.29% and 9.8% respectively.

**Discussion:**

The analytic performance of the FACSPresto against the reference standard was very good with better agreement and precision than the PIMA. The FACSPresto had comparable sensitivity at a threshold of 500 cells/μl and better specificity than the PIMA. However the FACSPresto showed reduced sensitivity at low CD4 count thresholds.

**Conclusion:**

The FACSPresto can be reliably used as a POC device for enumerating absolute CD4 count and CD4% values.

## Introduction

CD4+ T lymphocyte counts have been used to assess the risk of developing opportunistic infections, determine when to initiate ART and monitor the immunologic response to ART[1–3]. Access to CD4+ T lymphocyte testing in much of sub-Saharan Africa remains poor and can often be a significant barrier for ART treatment initiation[4]. Traditional methods of CD4 enumeration require centralized laboratories, often with expensive equipment, high maintenance costs and the need for skilled personnel[5]. Point of care tests provide an alternative by allowing for near patient testing, often with more affordable technologies, lower reagent and maintenance costs, and can be performed by individuals who do not have specialized training in laboratory techniques[6]. Licensed point of care (POC) CD4+T lymphocyte testing technologies include the PointCare NOW (PointCare Technologies), PIMA (Alere) and CyFlow miniPOC (Partec)[7]. The PointCare Now platform was previously shown to have poor analytic performance when compared with FACSCalibur. It had high relative bias particularly at CD4 counts below 350cells/μl and has not been adopted for routine clinical use[8]. The CyFlow miniPOC is based on flow cytometry and has been recently evaluated. Compared with the FACSCalibur (BD Biosciences) and FACSCount (BD Biosciences) as reference standards it showed low relative bias and good sensitivity at WHO defined thresholds[9]. The assay is performed on venous blood, has a rapid specimen processing time allowing for up to 250 patient tests/day and also enumerates CD4% values.

The PIMA is the only point of care device that is currently in widespread use in sub-Saharan Africa[10]. It is an automated image based instrument with lower absolute mean CD4 counts than the reference standards such as the FACSCalibur and FACSCount, but with good sensitivity and specificity at most CD4 thresholds[10–12]. The PIMA has played an important role in improving access to CD4 testing[13, 14], however it has certain limitations. The PIMA does not enumerate the CD4%, which is important for immunologic monitoring of pediatric patients on ART. In children below the age of 5 years, absolute CD4 counts rapidly change with age irrespective of ART while there is less perturbation of the CD4% with age. The analysis of a specimen on the PIMA takes about 20 minutes with sample and reagent incubations occurring in the instrument. This limits the number of samples that can be run each day to 20–25 specimens. A novel flow cytometry based device for enumerating the absolute CD4 count, CD4% and total hemoglobin concentration, the FACSPresto (Becton Dickinson (BD) Biosciences, NJ, USA) has recently been developed. It uses pre-prepared cartridges that contain CD4-PE-Cy^™^5, CD3-APC/CD45RA-APC/CD14-PE dried antibodies. Capillary or venous blood samples are transferred directly onto the cartridge and reagent and sample incubation occurs outside of the instrument allowing for analysis of up to 60 samples each day. The ability to provide both CD4% and hemoglobin will facilitate the management of both children and adults, and guide therapeutic options and patient management for those with abnormal hemoglobin levels.

We conducted an evaluation of the performance of the FACSPresto in CD4 and CD4% determination and used the FACSCalibur as the reference technology. We also compared the performance of the FACSPresto to the PIMA in a subset of patients.

## Methods

Blood samples were obtained from patients undergoing routine CD4 monitoring at Parirenyatwa Hospital Family care Centre (PHFCC). PHFCC is an HIV/ART clinic affiliated with the University of Zimbabwe College of Health Sciences (UZCHS). All analyses were conducted within research laboratories at UZCHS. All specimens were processed within 24 hours of collection. Blood was collected in K3-EDTA tubes and inverted several times to ensure proper mixing. For analysis on the FACSPresto analysis, a drop of blood from a Pasteur pipette was loaded onto the FACSPresto cartridge and capped and incubated at room temperature for 18 minutes; following incubation the cartridge was loaded onto the analyzer. For analysis on the PIMA, a drop of blood was loaded onto the cartridge and inserted into the analyzer.

The TruCount method on the FACSCalibur was performed as the reference standard. In brief, 20μl of BD Multitest fluorescent conjugated monoclonal antibodies, and 50μl of whole blood were added to the TruCOUNT tube and vortexed for 5 seconds. The Multitest consists of CD3-FITC/CD8-PE/ CD45-PerCP/CD4 APC reagent. The mixture was incubated for 15 min at room temperature in the dark before adding 450μl of FACS^™^ lysing solution and incubating for an additional 15 minutes in the dark prior to acquisition on the FACSCalibur. Data were analyzed using the MultiSET^™^ software using automated gating and analysis.

Analyses were conducted concurrently on the FACSCalibur, the FACSPresto and the PIMA in a subset of specimens. Triplicate analyses were conducted on all 3 platforms for 29 specimens.

The same operators for each instrument performed the immunostaining procedures and flow cytometry analyses. Each operator had received adequate training on the use of reverse pipetting technique. All operators received training on the performance of the assay by the manufacturer. A total of 3 operators were involved in the evaluation. Maintenance and instrument calibration was performed according to the manufacturers guidelines and done prior to initiation of the evaluation. Internal quality control was monitored routinely and the labs participated in an external quality assurance program with the Zimbabwe National Quality Assurance Program (ZINQAP). The lab that conducted the BD FACSCalibur subscribes to the College of American Pathologists proficiency-testing program for complete blood count (CBC) testing and United Kingdom National External Quality Assurance Service (UK-NEQAS) for CD4 testing. Samples are routinely tested for FBC and CD4 in parallel, this allows for internal quality control to be performed by comparing CD45+ (total lymphocytes) counts obtained from the FBC machine versus that obtained by BD Multitest CD3 FITC/CD8 PE/ CD45 Per-CP/CD4 APC reagent.

### Statistical methods

CD4+ T cell counts obtained from the FACSPresto and PIMA device were compared to the FACSCalibur and to each other. Descriptive statistics was used to describe the data. Differences in parameters between the two groups were determined by Wilcoxon signed rank test and paired t-test. Passing-Bablok regression was used for the method correlation and correlation coefficients were determined. To determine the bias between the platforms, Bland-Altman analysis was done[15]. The bias was defined as the mean difference between two methods. The limits of agreement were calculated as the mean±1.96 Standard Deviation (SD) of the differences of the results obtained. Confidence intervals for the bias and the limits of agreement were calculated. Pollock analysis was done to calculate the relative bias and the limits of agreement, which were defined as the mean±1.96SD of the relative mean bias of paired measurements. The data was plotted with the y-axis representing the % difference relative to the absolute value (x-axis) of the comparator test[16]. We determined the percentage similarity between a sample pair and defined it as the average between the methods divided by the comparator method multiplied by 100[17]. The same analysis was done for comparing CD4% values on the FACSPresto and the FACSCalibur.

The coefficient of repeatability was calculated and was defined as the variation in triplicate measurements for 29 specimens, performed on the same instrument by a single technician under the same conditions. Coefficient of repeatability below 5% were considered optimal, coefficients between 5–10% were considered acceptable[18, 19].

Data was stratified into two groups based on CD4 count above or below 350 cells/μl and 500 cells/μl and CD4% above or below 25%. Agreement between the results obtained on the different platforms at CD4 counts below thresholds of 350 cells/μl and 500 cells/μl was determined using kappa statistics. We applied the Landis-Koch interpretation scale (kappa values of <0.40 indicate poor agreement; >0.40 and <0.75 fair to good agreement and >0.75 excellent agreement).

### Ethical Review

The Joint Research and Ethics Committee of the University of Zimbabwe College of Health Sciences and Parirenyatwa Hospital, the Medical Research Council of Zimbabwe and Partners HealthCare Human Research Committee approved the protocol prior to implementation. Blood samples were collected in the context of routine CD4 count testing for both ART naïve and experienced patients at Parirenyatwa Hospital Family Care Centre (PHFCC). PHFCC is a public HIV clinic under the Ministry of Health and Child Care (MOHCC). Written informed consent is not required for receipt of routine services including CD4 testing performed at MOHCC facilities. Residual blood from routine testing was used for the analysis. No personally identifiable information was made available to the researchers. The institutional review boards waived the need for written informed consent.

## Results

Specimens collected from 253 patients were tested on the FACSCalibur, and FACSPresto. There were 3 failed runs on the FACSPresto. Thus the final analysis was done on 250 specimens. CD4+ T cell values ranged from 58 to 1275 cells/μl on the FACSCalibur, and 68 to 1257 cells/μl on the FACSPresto. The median CD4 counts on the FACSCalibur and FACSPresto were 501 cells/μl (IQR: 328, 623), 514 cells/μl (IQR: 334, 696) respectively (p = 0.0013). Among the specimens, 50% (n = 125) were ≤500 cells/μl and 29% (n = 73) were ≤350 cells/μl on the FACSCalibur.

**Fig 1:**
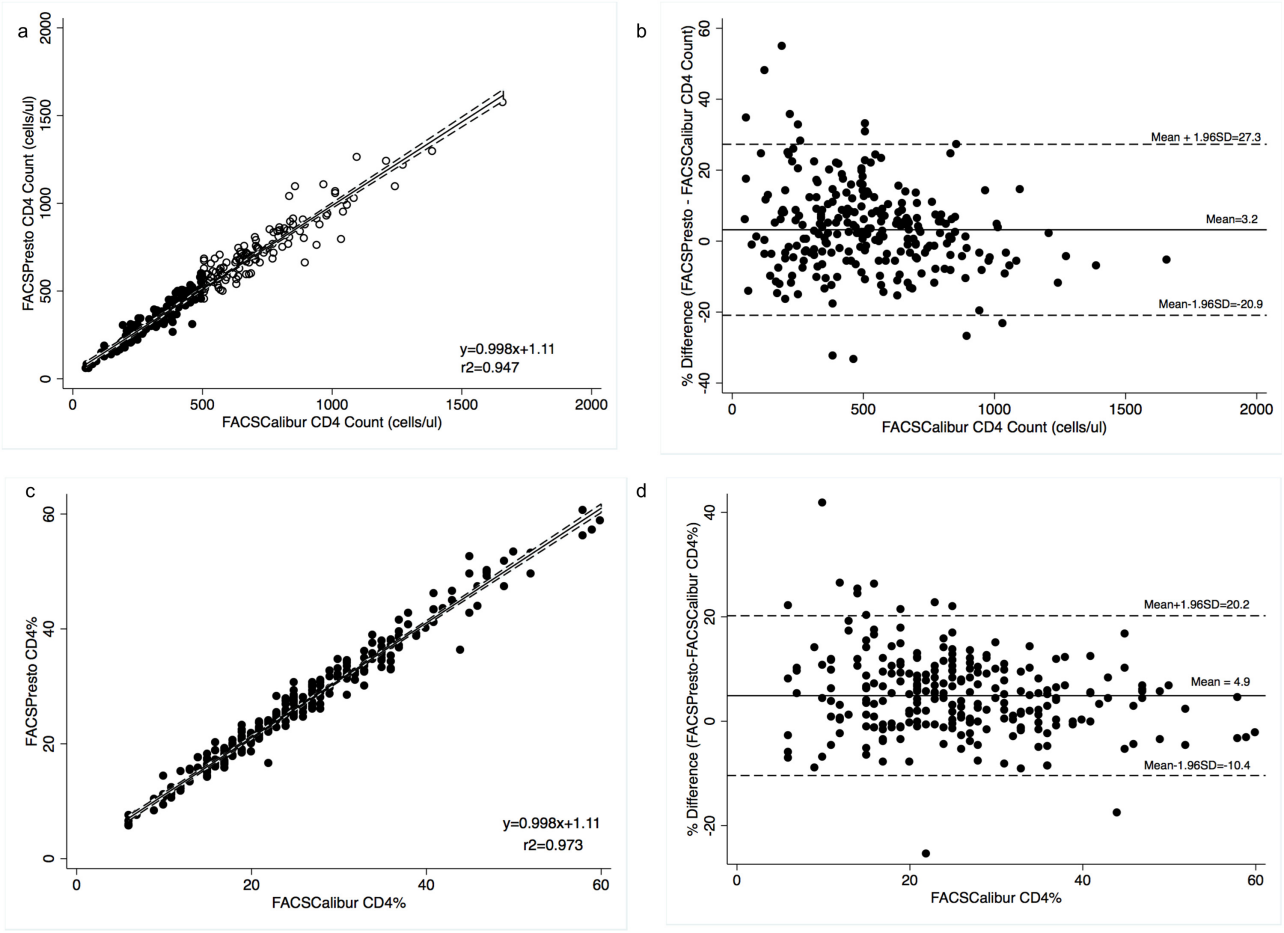
Comparison between FACSPresto and FACSCalibur. Passing-Bablok regression plot comparison of (a) absolute CD4 count and (c) CD4% values obtained from FACSPresto with the FACSCalibur as reference standard. The solid line represents the regression line and dashed line the 95%CI. Pollock plots indicating %mean bias between (b) absolute CD4 count and (d) CD4% values obtained on FACSPresto compared with those obtained on the FACSCalibur. The solid line represents the mean bias, the dashed line represents mean bias ±1.96SD.

**Bias analysis for absolute CD4 count enumeration on the FACSPresto and FACSCalibur.** The mean absolute bias between the FACSPresto and FACSCalibur values was 9.5 cells/μl (p = 0.0148)(Table 1). The 95% limits of agreement (LOA) were -110 cells/μl and 130 cells/μl (Table 1). The relative mean bias (mean% bias) was 3.2%; the 95% LOA were -20.9% and 27.3% (Fig 1b). There was a significant difference in mean % bias for thresholds of 350 cells/μl (5.7% vs. 2.2%, p = 0.038) and 500 cells/μl (4.8% vs. 1.6%, p = 0.0427) suggesting improved agreement at higher CD4 counts. Among the specimens 64% of values obtained on the FACSPresto were within ±10% of the values obtained on the FACSCalibur. The mean percentage similarity was 101.6% (coefficient of Variation (CV) 6%). There was a significant difference in % similarity at threshold of 350 cells/μl (102.9% and 101.1%, p = 0.038) and 500 cells/μl (102.4% and 100.8%, p = 0.0427). The coefficients of determination (r^2^) were 0.947 for all specimens (Fig 1a) and 0.882 and 0.899 for CD4 ≤ 350 cells/μl and CD4> 350 cells/μl respectively, and 0.899 and 0.858 for CD4 ≤ 500 cells/μl and CD4> 500 cells/μl respectively (Table 1).

**Table 1:** Median CD4 count and measures of bias between reference method and FACSPresto or PIMA.

CD4 Platform	Reference	N	Median CD4 (IQR)		p-value	R2	Mean Bias (LOA)	Mean% bias (SD)	Mean % similarity (%CV)
			Platform	Reference					
**FACSPresto**	**FACSCalibur**	250	514 (334, 696)	501 (328, 683)	0.0013	0.947	9.5 (-110.9–130.0)	3.2 (12.3)	101.6 (6.0)
CD4≤350 cells/μl	FACSCalibur	73	266 (173, 307)	239 (184, 315)	0.0077	0.882	12.2 (-48.7–73.3)	5.7 (14.7)	102.9 (7.2)
CD4>350 cells/μl	FACSCalibur	177	612 (500, 751)	591 (493, 764)	0.0359	0.899	8.4 (-129.4–146.2)	2.2 (11.0)	101.1 (5.5)
CD4≤500 cells/μl	FACSCalibur	125	334 (237, 436)	328 (225, 409)	0.0001	0.899	13.8 (-67.5–95.0)	4.8 (13.8)	102.4 (6.7)
CD4>500 cells/μl	FACSCalibur	125	696 (593, 833)	683 (575, 835)	0.337	0.858	5.3 (-144.4–155.0)	1.6 (10.4)	100.8 (5.2)
**PIMA**	**FACSCalibur**	41	416 (230, 666)	490 (254, 764)	<0.0001	0.938	-68.4 (-225.7–88.8)	-10.5 (17.6)	94.8 (9.3)
CD4≤350 cells/μl	FACSCalibur	13	200 (169, 220)	207 (125, 248)	0.133	0.628	-19.8 (-121.1–81.5)	-3.4 (24.5)	98.3 (12.4)
CD4>350 cells/μl	FACSCalibur	28	569 (409, 712)	680 (484, 834)	<0.0001	0.902	-91 (-251.3–69.3)	-13.8 (12.6)	93.1 (6.7)
CD4≤500 cells/μl	FACSCalibur	22	234 (198, 339)	290 (205, 406)	0.017	0.795	-30.9 (-152.9–91.2)	-6.8 (21.7)	96.6 (11.2)
CD4>500 cells/μl	FACSCalibur	19	667 (516, 803)	775 (677, 899)	0.0002	0.903	-111.9 (-264.6–40.7)	-14.7 (10.3)	92.6 (5.6)
**PIMA**	**FACSPresto**	41	416 (230, 666)	500 (302, 762)	<0.0001	0.936	-77.7 (-233.0–77.6)	-14 (12.4)	93.0 (6.7)
CD4≤350 cells/μl	FACSPresto	14	203 (169, 230)	236 (171, 302)	0.011	0.838	-32.6 (-106.1–40.8)	-11.1 (13.3)	94.4 (7.0)
CD4>350 cells/μl	FACSPresto	27	574 (416, 736)	710 (500, 856)	<0.0001	0.892	-102 (-268.2–64.1)	-15.5 (11.9)	92.3 (6.5)
CD4≤500 cells/μl	FACSPresto	21	230 (198, 329)	302 (196, 413)	0.0003	0.895	-50.4 (-143.4–42.5)	-14.2 (13.0)	92.9 (7.0)
CD4>500 cells/μl	FACSPresto	20	667 (540, 795)	782 (657, 874)	0.0006	0.842	-106.3 (-294.0–81.4)	-13.8 (12.1)	93.1 (6.5)
**FACSPresto**	**FACSCalibur**	250	25.7 (18.4, 32.9)	25 (17, 33)	<0.0001	0.973	1.06 (-2.5–4.7)	4.9 (7.8)	102.4 (3.8)
CD4≤25%	FACSCalibur	139	17.8 (14.5)	19 (15, 22)	<0.0001	0.938	1.14 (-1.8–4.1)	6.4 (8.7)	103.2 (4.2)
CD4>25%	FACSCalibur	111	32.5 (27.9)	33 (28, 39)	<0.0001	0.931	0.97 (-3.3–5.3)	2.9 (6.1)	101.5 (3.0)

**Bias analysis for CD4% enumeration on the FACSPresto and FACSCalibur.** The CD4% values ranged from 6% to 58% on the FACSCalibur and 5.83% to 57.1% on the FACSPresto. The mean bias between the FACSPresto and FACSCalibur values for CD4% was 1.06% (p = 0.46) (Table 1) and the 95%LOA were -10.4% and 20.2% (Fig 1d). The relative mean bias (mean% bias) was 4.9% and the 95% limits of agreement (LOA) were -10.4% and 20.2% (Table 1). Among the specimens, 76% of CD4% values obtained on the FACSPresto were within ±10% of the values obtained on the FACSCalibur. There was a significant difference in relative mean bias at CD4≤25% and CD4>25% (6.4%vs. 2.9%, p = 0.0004), suggesting better agreement at higher CD4%. The mean % similarity was also better at higher (CD4>25%) compared to lower (CD4≤25%) CD4% thresholds (101.5 vs. 103.2, p = 0.0004). The coefficients of determination (r^2^) were 0.973 for all specimens (Fig 1c) and 0.938 and 0.931 for CD4 ≤ 25% and CD4> 25% respectively. (Table 1).

**Fig 2:**
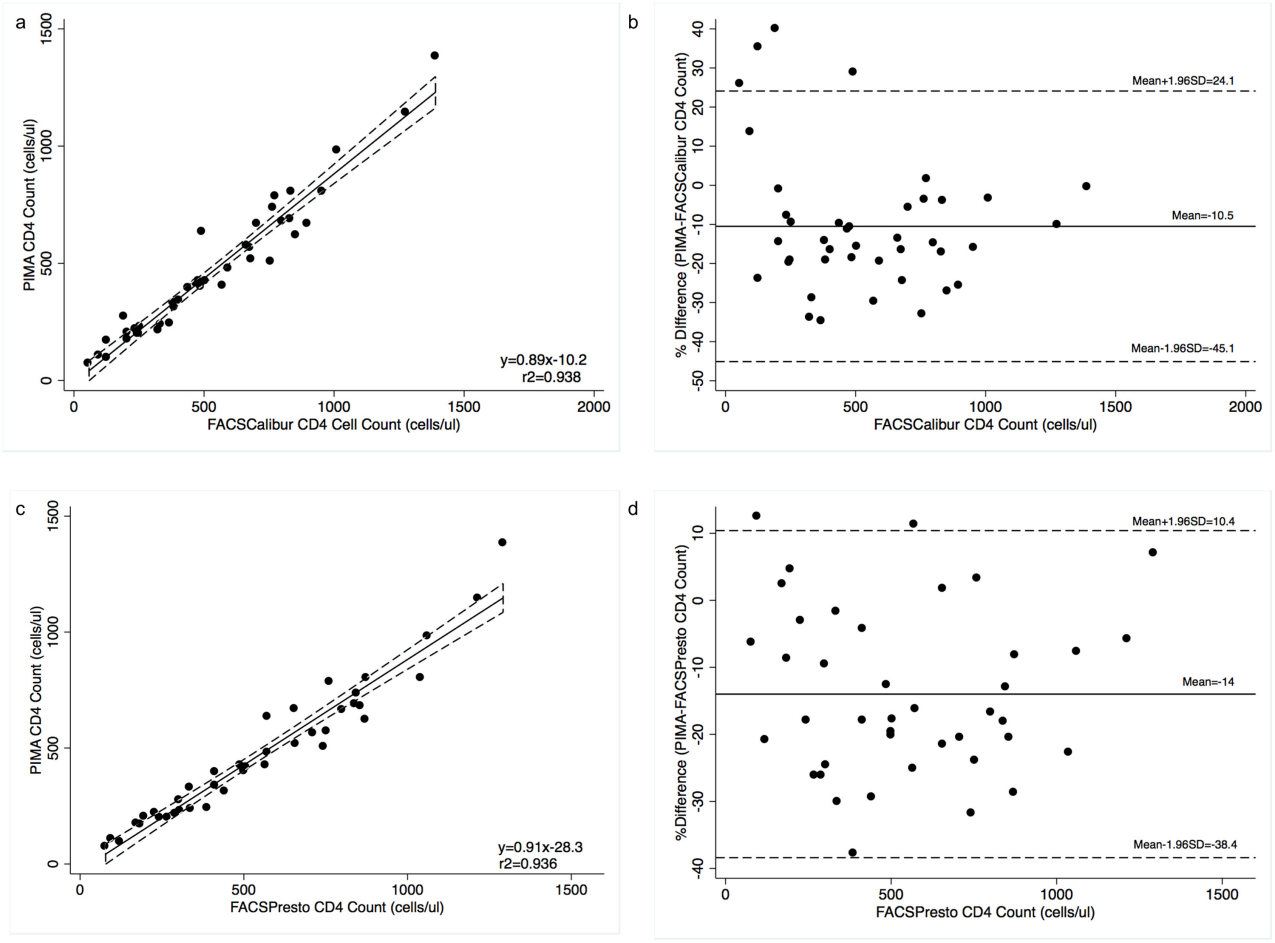
Comparison between PIMA and FACSCalibur and FACSPresto. (a) Passing-Bablok regression plot comparison of absolute CD4 count between PIMA and FACSCalibur; (b) Pollock plot indicating %mean bias between PIMA and FACSCalibur

**Bias analysis for absolute CD4 count enumeration on the PIMA and FACSCalibur.** The point of care instrument that is in widespread use in sub-Saharan Africa is the PIMA. We compared the PIMA to the reference method FACSCalibur as well as to the FACSPresto. A total of 41 specimens were additionally tested on the PIMA platform. The range of CD4 counts tested was 73 to 1382 cells/ μl on the PIMA. The absolute CD4 values on the PIMA were on average lower than those on the FACSCalibur. The absolute mean bias between the PIMA and FACSCalibur values was -68.4 cells/μl (p<0.0001)(Table 1). The 95% limits of agreement (LOA) were -225.7cells/μl and 88.8 cells/μl (Table 1). The relative mean bias (mean% bias) was -10.5%; the 95% LOA were -45.1% and 24.1% (Fig 2b). There was no significant difference in mean % bias for thresholds of 350 cells/μl (-3.4% vs. -13.8%, p = 0.0787) and 500 cells/μl (-6.8% vs. -14.7%, p = 0.156). Among the specimens only 24.4% of values obtained on the PIMA were within ±10% of the values obtained on the FACSCalibur. The mean percentage similarity was 94.8% (CV 9.3%), reflecting the generally lower values obtained on the PIMA. The difference in % similarity at thresholds of 350 cells/μl (98.3% and 93.1%, p = 0.0787) and 500 cells/μl (96.6% and 92.6%, p = 0.1157) was not significant. The coefficients of determination (r^2^) were 0.938 for all specimens (Fig 2a) and 0.628 and 0.902 for CD4 ≤ 350 cells/μl and CD4> 350 cells/μl respectively, and 0.795 and 0.903 for CD4 ≤ 500 cells/μl and CD4> 500 cells/μl respectively (Table 1).

Absolute CD4 count values; (c) Passing-Bablok regression plot comparison of absolute CD4 count between PIMA and FACSPresto; (d) Pollock plot indicating %mean bias between PIMA and FACSPresto. In Passing-Bablok plots the solid line represents the regression line and dashed line the 95%CI. In the Pollock plots the solid line represents the mean % bias, the dashed line represent mean bias ±1.96SD.

The absolute CD4 values on the PIMA were also lower compared with those on the FACSPresto. The mean absolute bias between the PIMA and FACSPresto values was -77.7 cells/μl (p<0.0001)(Table 1). The 95% limits of agreement (LOA) were -233 cells/μl and 77.6 cells/μl (Table 1). The relative mean bias (mean% bias) was -14%; the 95% LOA were -38.4% and 10.4% (Fig 2d). There was no significant difference in mean % bias for thresholds of 350 cells/μl (-11.1% vs. -15.5%, p = 0.295) and 500 cells/μl (-14.2% vs. -13.8%, p = 0.922). Among the specimens only 34.1% of values obtained on the PIMA were within ±10% of the values obtained on the FACSPresto. The mean percentage similarity was 93.0% (CV 6.7%), again reflecting the generally lower values obtained on the PIMA. There was a small non-significant difference in % similarity at threshold of 350 cells/μl (94.4% and 92.3%, p = 0.295) and 500 cells/μl (92.9% and 93.1%, p = 0.09224). The coefficients of determination (r^2^) were 0.936 for all specimens (Fig 2c) and 0.838 and 0.892 for CD4 ≤ 350 cells/μl and CD4> 350 cells/μl respectively, and 0.895 and 0.842 for CD4 ≤ 500 cells/μl and CD4> 500 cells/μl respectively (Table 1).

**Precision analysis.** We determined the precision of the assays on the different instruments by taking triplicate measurements of each sample on each instrument with the same operator and determining the coefficients of variation. The coefficients of repeatability on the BD FACSCalibur, FACSPresto, and PIMA were 4.13%, 5.29%, and 9.79% respectively.

**Determining Eligibility for ART.** We determined the level of agreement and sensitivity and specificity of each platform in classifying patients at thresholds of ≤350cells/μl and ≤500 cells/μl with the FACSCalibur as the reference standard. At a threshold of ≤350 cells/μl there was 93.2% agreement (k = 0.832). The sensitivity and specificity, PPV and NPV of the FACSPresto was 84.9%, 96.6%, 91.2% and 94% respectively (Table 2). At a threshold of ≤500 cells/μl there was 94.8% agreement (k = 0.896). The sensitivity improved with a higher treatment threshold. The sensitivity and specificity, PPV and NPV of the Presto was 92.8%, 96.8%, 96.7% and 93.1% respectively. The downward misclassification rate at a threshold of ≤350cells/μl was 3.2% and upward misclassification rate was 15.1%. This would result in 15.1% inappropriately classified as having a CD4>350 resulting in a delay in ART initiation at those thresholds. The upward misclassification rate decreased to 7.2% using a higher treatment initiation threshold of ≤500cells/μl (Table 2).

**Table 2:** Sensitivity, Specificity, Positive (PPV) and Negative Predictive values (NPV) and misclassification rates of absolute CD4 counts at thresholds of 350 cells/μl and 500 cells/μl for FACSPresto and PIMA with FACSCalibur as the reference standard.

Platform	Threshold	Sensitivity (95% CI)	Specificity (95% CI)	PPV	NPV	Downward Misclassific-ation	Upward Misclassifi-cation
**FACSPresto**	350 cells/μl	84.9% (74.6–92.2)	96.6% (92.8–98.7)	91.2% (81.8–96.7)	94% (89.4–96.9)	3.40%	15.10%
	500 cells/μl	92.8% (86.8–96.7)	96.8% (92–99.1)	96.7% (91.7–99.1)	93.1% (87.3–96.8)	3.20%	7.20%
**PIMA**	350 cells/μl	100% (75.2–100)	85.7% (67.3–96)	76.5% (50.1–93.2)	100% (85.8–100)	14.30%	0%
	500 cells/μl	95.5% (77.2–99.9)	84.2% (60.4–96.6)	87.5% (67.6–97.3)	94.1% (71.3–99.9)	15.80%	4.50%

There was slightly less agreement between the PIMA and the FACSCalibur than that between the FACSPresto and the FACSCalibur. The agreement between the PIMA and the FACSCalibur at threshold of ≤350cells/μl was 90.2% (k = 0.792) and 90.4% (k = 0.802) at a threshold of ≤500cells/μl. The sensitivity and specificity, PPV and NPV of the PIMA compared with the BD FACSCalibur as reference at a threshold of ≤350cells/μl, was 100%, 85.7%, 76.5% and 100%. At a threshold of ≤500cells/μl the sensitivity and specificity, PPV and NPV was 95.5%, 84.2%, 87.5% and 94.1% respectively. The PIMA had better sensitivity when compared with the FACSPresto, however specificity was poorer. The downward misclassification rates were 14.3% and 15.8% at thresholds of ≤350cells/μl and ≤500cells/μl respectively. This would result in significant proportions of individuals with high CD4 counts classified as having low CD4 counts requiring initiation of ART. With the increasing trend to initiate individuals at higher CD4 counts due to improved mortality and morbidity, the clinical significance of this misclassification is likely minimal.

## Discussion

Point of care tests have been shown to accelerate time to initiation of ART and increase the proportion of individuals initiating ART[13, 14] and may be a cost-effective way to provide HIV care[20]. We evaluated the analytic performance of the FACSPresto a novel point of care device. The results obtained were comparable to those that have been obtained in comparisons between the FACSCalibur and the FACSCount which was the first single platform cytometer dedicated to CD4 testing[7, 19, 21]. The FACSCount is in widespread use in many central laboratories in sub-Saharan Africa and is often used as the reference standard for evaluating new CD4 technologies[7]. The relative mean bias and similarity between FACSCount and FACSCalibur in a multisite WHO study was 3.1% and 102% respectively[21]. We found similar results in comparing the FACSPresto to the FACSCalibur. The relative mean bias was 3.2% and percentage similarity was 101.6%, suggesting that these platforms could be used interchangeably. The mean absolute and relative (mean %) biases between the FACSPresto and the FACSCalibur were slightly higher for specimens with lower CD4 counts than those with higher values. This phenomenon has been shown in other method comparison studies of CD4 testing technologies leading to lower sensitivity at low CD4 count thresholds[10].

Over the years thresholds for ART initiation have shifted from <200 cells/μl, to <350 cells/μl in 2010 and to <500 cells/μl in 2013 WHO guidelines[22, 23]. More recently, in response to randomized trials showing significant morbidity and mortality benefits with ART initiation at CD4 counts >500 cells/μl, guidelines are shifting towards recommending ART initiation for all ages regardless of CD4+ T cell count[24, 25]. Many countries have adopted the 2013 WHO guidelines however several continue to use thresholds of 350 cells/μl[26]. In a previous comparison of the sensitivity and specificity of the reference standards the FACSCount and FACSCalibur at a threshold of ≤350 cells/μl and ≤500 cells/μl sensitivities and specificities were slightly lower at the lower CD4 count threshold[21]. In this evaluation the sensitivity of the FACSPresto was also lower at a CD4 count threshold of ≤350 cells/μl when compared with ≤500 cells/μl. The low sensitivity led to a high upward misclassification rate that would have important clinical implications, as individuals who need to start ART would be delayed in ART initiation. This may become less relevant as CD4 counts play a less significant role in determining the threshold for ART initiation. However CD4 counts will continue to play an important role as a prognostic marker and in determining when to implement screening for conditions such as Cryptococcal meningitis. The risk of cryptococcal disease increases at a CD4 count threshold of ≤100 cells/μl[27] and screening with pre-emptive treatment may have an impact on outcomes[28]. The increased bias, and low sensitivity at lower CD4 count thresholds raises some concerns as this may be further exacerbated at thresholds of ≤100 cells/μl. Our study sample and that of another recent evaluation[29] have all had insufficient representation of specimens with CD4 counts below 100 cells/μl, reflecting the decreased incidence of severe immunosuppression within clinical cohorts. However, a dedicated study evaluating performance at very low thresholds is required to help inform programs that are implementing screening for cryptococcal disease and POC CD4 testing.

In the children below the age of 5 years, there is significant variability in absolute CD4 count values while CD4% values are less perturbed by age. The CD4% is used in pediatric HIV infection as a marker of the severity of immunosuppression. The FACSPresto unlike the PIMA enables the assessment of CD4% in pediatric patients. In this evaluation CD4% values on the FACSPresto were comparable to those on the FACSCalibur with a low mean bias. In 2010 and 2013 WHO guidelines began to expand ART initiation criteria to ART initiation for all children below age 5 years irrespective of absolute CD4+ T cell count or CD4%[30]. This was in recognition of the morbidity and mortality benefits of early ART in children[31]. This removed CD4 count testing as a barrier to ART initiation however it did not remove the significance of testing as a prognostic factor[32]. In the future in both adult and pediatric HIV treatment and care, absolute CD4 count and CD4% are likely to continue to play a role in baseline care as well as in the setting of virologic failure as prognostic markers of disease progression while also guiding initiation of preventive treatment strategies for opportunistic infections.

We compared the results on the FACSPresto to those obtained on another POC device, the PIMA. The PIMA has been evaluated extensively. In a large pooled analysis, PIMA values were consistently lower than those from reference technologies[10]. In that analysis the sensitivity at a threshold of 350cells/μl was comparable to that at a threshold of 500 cells/μl (93.4% vs. 96.9%), the specificity was slightly lower at both thresholds (89.1% vs. 81.3% respectively)[10]. We also found that the sensitivity was good at both thresholds (100% vs. 95.5%) and the specificity was considerably lower than the sensitivity at both thresholds (85.7% and 84.2%). This poor specificity leads to high downward misclassification rate in the PIMA relative to the reference standard which was not evident with the FACSPresto which had specificities of >96% at all thresholds. The clinical impact of this relatively higher downward misclassification rate on long-term outcomes is likely minimal given the benefits of early ART initiation[24, 25]. The only exception is if this extends to even lower thresholds of CD4 counts ≤100cells/μl for screening for cryptococcal disease, as this would result in a larger proportion of individuals inappropriately screened for disease with implications on screening costs.

The FACSPresto performed well and can serve as a replacement technology for more expensive, technically more challenging reference instrument such as the FACSCalibur. We found the instrument reliable, simple to use, and with a coefficient of repeatability that was comparable to that of the FACSCalibur[33]. The FACSPresto meets many of the criteria for a good point of care diagnostic test for resource limited settings. It has a high sensitivity and specificity in comparison with a known reference standard, it is easy to use by non-technical staff, it is rapid and uses reagents that do not require refrigerated storage. The off-board reaction allows the test to process more samples per day than the PIMA and may facilitate improved clinic workflow particularly in busy settings.

A limitation of our study is that we did not test the performance on capillary blood samples in the field. Capillary blood in the field may result in more errors due to insufficient quantity and greater variability based on technique used to obtain the sample. In addition pediatric samples were not available for this analysis. However our data adds to the field by evaluating a novel POC device that has distinct advantages over existing POC technologies, is reliable and is comparable with a reference technology. In addition we compared it to a POC technology that is in routine use in much of sub-Saharan Africa. Although virus load testing is anticipated to eclipse CD4 counts in long-term monitoring, at present CD4 testing at POC with rapid turn-around time informs opportunistic infections prophylaxis, ART management and may guide switching to second line therapy or prompt targeted virus load testing[7, 34]. Improved access to POC CD4 testing remains important, and the good accuracy and performance characteristics of the FACSPresto make it a suitable alternative to the reference standard technologies.
